# Maxillary labial peri-implant hard and soft tissue alteration observed on cross-sectional dimension: a 2-year prospective observational study

**DOI:** 10.1186/s40729-023-00477-z

**Published:** 2023-06-23

**Authors:** Shuhei Yamada, Tamaki Nakano, Tomoyuki Kobayashi, Shoichi Ishigaki

**Affiliations:** Department of Fixed Prosthodontics and Orofacial Function, Osaka University Graduate School of Dentistry, 1-8 Yamadaoka, Suita, Osaka 565-0871 Japan

**Keywords:** Platform switching implants, Prospective study, Observational study, Peri-implant hard and soft tissue alteration, Peri-implant tissue classification

## Abstract

**Objectives:**

To evaluate how peri-implant hard and soft tissue height (BH, MH) alter after final prostheses placement related to labial hard and soft tissue thickness (BW, MW).

**Materials and methods:**

Forty-five platform-switched implants were classified into four groups according to BW and MW: type 1 (thick BW and thick MW), type 2 (thick BW and thin MW), type 3 (thin BW and thick MW), type 4 (thin BW and thin MW). Tissue resorption was evaluated on cone-beam CT images taken at final prostheses placement, at 1-year follow-up, and at 2-year follow-up. Kruskal–Wallis test and post hoc Mann–Whitney test were applied; significance was set to 0.05.

**Results:**

BH resorption was 0.13 ± 0.12 mm in type 1, 0.26 ± 0.17 mm in type 2, 0.09 ± 0.09 mm in type 3, 0.94 ± 0.19 mm in type 4. Differences between type 1 and 4, type 2 and 4, and type 3 and 4 were statistically significant (*p* < 0.001, *p* = 0.005, *p* < 0.001, respectively). MH resorption was 0.10 ± 0.09 mm in type 1, 0.36 ± 0.16 mm in type 2, 0.12 ± 0.12 mm in Type 3, 0.79 ± 0.23 mm in type 4. Differences between type 1 and 2, type 1 and 4, type 2 and 3, type 2 and 4 and type 3 and 4 were statistically significant (*p* < 0.001).

**Conclusions:**

Significantly less BH/MH resorption occurs around implants with thick BW/MW than those with thin BW/MW in 2 years. Implants with thick peri-implant soft tissue resulted in significantly less tissue resorption in second year after final prostheses placement.

## Introduction

Dental implant therapy in the maxillary anterior region aims not only for functional aspect but also esthetic aspect for optimizing patient benefits, achieving peri-implant tissue condition in harmony with the remaining natural dentition. Peri-implant soft tissue recession is one of the complications which could occur over time after implant superstructure placement [1]. Sufficient labial/buccal peri-implant hard and soft tissue is one of the prerequisites for constructing resistant conditions of peri-implant tissue against tissue resorption in time. [2]

It has been reported that the presence of hard tissue with a thickness of at least 2.0 mm on the labial/buccal aspect of the implant body is required for the long-term stability of peri-implant tissue [2]. Merheb et al. also reported that obtaining at least 2.0 mm of buccal bone around the implant would prevent vertical resorption [3]. Spray et al. reported that implants with 1.8 mm or more buccal bone showed less bone resorption [4]. Suppose the pre-operative examination indicates that labial/buccal hard tissue is insufficient, and the thickness of the implant body’s labial or buccal hard tissue is expected to be less than 1.5 mm. In that case, bone reconstructive surgery is often conducted on implant placement surgery.

On the other hand, it is reported that if labial or buccal soft tissue of implant is thin, resorption of the labial tissue is likely to occur after implant superstructure placement. Eventually esthetics will be impaired due to possible peri-implant mucosa recession [5]. Proposed indices of labial or buccal peri-implant soft tissue amount for peri-implant tissue stability vary from 1.0 mm, [6] 1.5 mm, [7] to 2.0–3.0 mm [8]. The inconclusiveness of a clinical indicator for peri-implant soft tissue thickness is considered problematic [9].

Most clinical indices for peri-implant soft tissue were referred to as the labial thickness of gingiva around natural dentition [10–12]. However, these indices are considered insufficient for preserving peri-implant tissue because of the lack of analyses on chronological peri-implant soft tissue alteration and the histological difference between periodontal and peri-implant tissue [13]. Several studies [14–16] analyzed how vertical soft tissue thickness correlates with marginal bone loss over time, lacking examination on labial/buccal tissue changes and follow-up term was limited within 1 year. Another study analyzed the association between buccal mucosa thickness and peri-implant bone loss in 7.65 ± 4.3 years. It concluded that there was no statistical correlation between them, lacking information on buccal/labial bone alteration [17].

The term “periodontal phenotype,” which signifies both the gingival phenotype and the thickness of the buccal bone plate, is then applied to “peri-implant phenotype” [18]. Surprisingly, no clinical studies are available until now that categorize implants according to labial/buccal hard and soft tissue thickness. However, as stated above, histological differences between natural dentition and implant must be considered, and the categorization in peri-implant tissue phenotype should be cautiously applied.

Therefore, this study aimed to investigate how peri-implant tissue alters after implant superstructure placement by categorizing implants according to labial/buccal hard and soft tissue thickness.

## Material and methods

### Study design and participants

In this prospective observational study, 31 patients (mean age: 57.5 years, range 19 to 74 years) participated in the present study who were partially edentulous and required prosthetic treatments in the maxillary anterior and premolar region at Osaka University Dental Hospital from June 2013 to August 2019. Each patient was given a detailed description of the study design and clinical procedures and was required to sign an informed consent before participation. The protocol for this study was conducted in agreement with the Declaration of Helsinki, considering the checklist items as proposed in the STROBE statement for cohort studies. Osaka University Institutional Review Board approved the study (registration number H30–E4). The power analysis using G*Power software (v 3.1.9.6.) determined the sample size. Due to the lack of existing clinical studies analyzing labial tissue thickness impact on peri-implant tissue resorption, a large effect size (*f* = 0.57) was assumed. A type I error rate of *α* = 0.05 was set. To achieve a power of at least *n* = 10 per group was obtained. Subjects for the present study were selected from partially edentulous patients who required implant-supported prostheses in the maxillary anterior or premolar region at Osaka University Dental Hospital from June 2013 to August 2019. The participants were recruited consecutively recruited during the presented observational period.

Inclusion criteria for the present study were as follows: (a) 18 years old or older; (b) in general good health condition; (c) absence of systemic diseases which affect bone metabolism and wound healing; (d) capable of willingly participating in the study; (e) platform-switched implants were placed; and (f) written informed consent to receive cone-beam CT scans at follow-ups obtained.

The subjects were not included in the present study if they present one of the following conditions: (a) diabetic patients (HbA1c > 7.5%); (b) smokers; (c) patients who were at the period of pregnancy or lactation at any time during the study; and (d) regular intake of medication which brings periodontal inflammation.

### Clinical procedure

Following clinical and radiographical examinations, implants with platform switched connections were placed by two-staged protocols by two experienced surgeons. Implants (NobelActive®/NobelReplace Tapered CC®, Nobel Biocare, Gothenberg, Sweden, Bone Level Implant®/Bone Level Tapered Implant®, Straumann, Basel, Switzerland) were placed acquiring adequate primary stability (insertion torque more than 20 Ncm). The implants were placed 4 mm apically to the prospective implant restoration using surgical templates. The implant body was selected according to ridge morphology and prosthetic design. Guided bone regeneration (GBR) procedure was performed when dehiscence on implant surface was present. Connective tissue grafting (CTG) procedure was also conducted upon second surgery when contour augmentation was required to achieve esthetic results. Among 45 implants in 31 patients, nine implants were placed without any hard and soft tissue augmentation, 21 implants were placed with guided bone regeneration, nine implants were placed with GBR and CTG, six implants were placed under immediate implant placement protocol, accompanied by simultaneous GBR procedures. GBR protocols were carried out using deproteinized bovine bone graft material (Bio-Oss®, Geistlich, Volhusen, Switzerland) and resorbable membrane (Bio-Gide®, Geistlich, Volhusen, Switzerland). Connective tissue grafts were harvested from the palate. After 6 months of healing, second surgery was performed, followed by 2 months of provisional restoration installations. Porcelain-fused-to zirconia crowns were cemented to titanium abutment interface, and access holes were drilled after cementation. Then, restorations were screwed to implants with 35 Ncm installation torque. Autoclaved PTFE tapes (Iso Tape, TDV, Pomerode, Brazil) were inserted into access holes, and composite resin was filled and polished thoroughly.

### CBCT image acquisition

Cone-beam CTs were scanned using Alphard 3030 (Asahi Roentgen Industry, Kyoto, Japan) at the time of final prostheses placement (T1), 1-year follow-up (T2), and 2-year follow-up (T3). Cotton roles were inserted in vestibules during CBCT scans to prevent labial and buccal soft tissue from migrating into the region of interest [19]. CBCT scans were taken with the following technical parameters: 80 kV acceleration voltage; 7 mA beam current; 833 cm [3] field of view (FOV) volume; 0.2 mm voxel size; and 17 s of scanning time.

Three dimensional (3D) maxillary models were reconstructed from DICOM data of each CBCT scan from T1 to T3 using a software (coDiagnostiX®, Dental Wings, Montréal, Canada). A virtual implant image was placed according to the reference of a subjected implant on CBCT of T1. The 3D maxillary models were then superimposed by utilizing certain reference points on jaw bones, enabling the virtual implant to appear on exactly the same position in CBCT of T2 and T3. Cross-sectional images on objected implants were obtained in the following manner; dental arch which follows the very center of both objected implant and remaining dentitions on a transverse plane of a CBCT scan, then a cross-sectional plane which was perpendicular to the established dental arch.

### Radiographic measurement

**Fig. 1:**
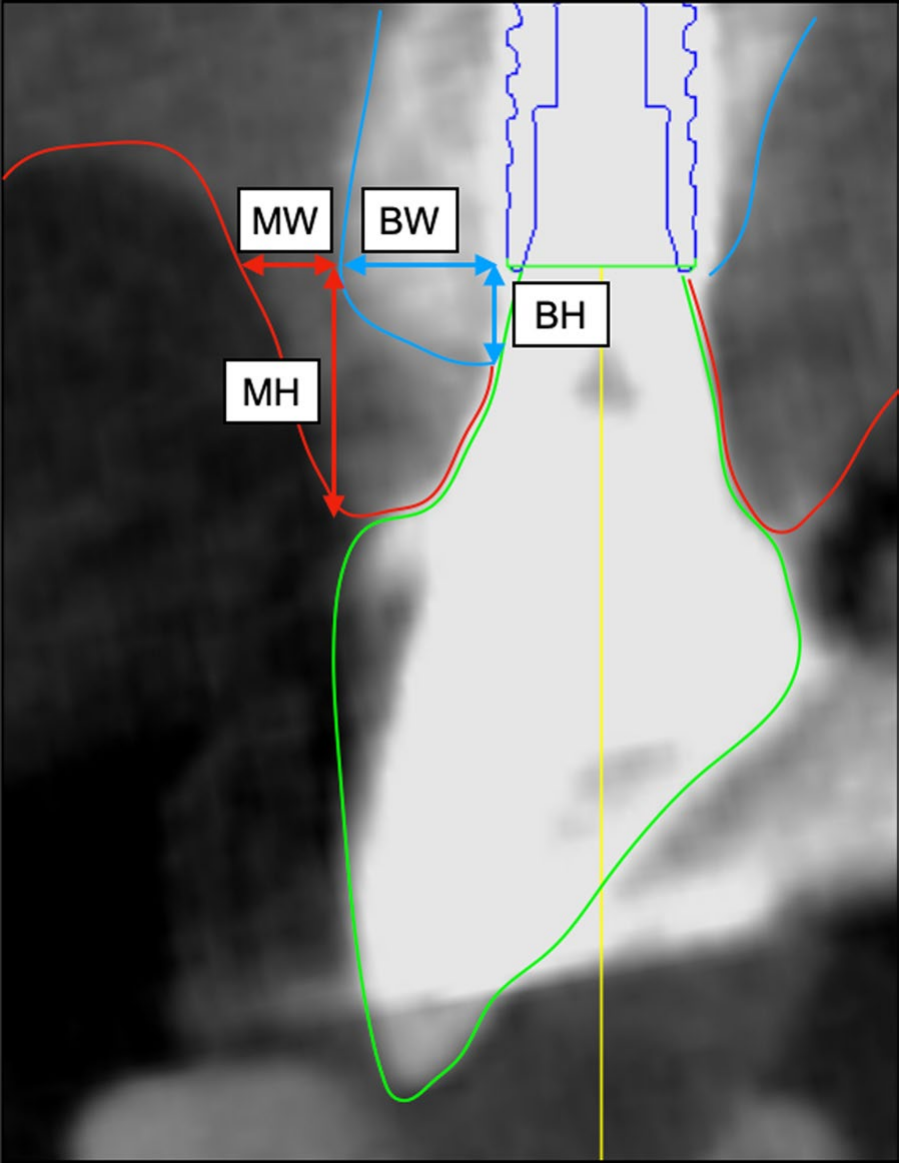
Representative images of examined implants with CBCT scans. Hard tissue width and soft tissue width of labial/buccal aspect on implant platform was measured as BW and MW. Hard tissue height and soft tissue height on labial/buccal aspect of implants were also measured as BH and MH. *BW* bone width, *MW* mucosa width, *BH* bone height, *MH* mucosa height

Hard tissue width (BW) and soft tissue width (MW) of the labial/buccal aspect of the implant platform were measured on acquired cross-sectional CBCT images. On the labial/buccal aspect of implants, hard tissue height and soft tissue height were also measured on cross-sectional CBCT images. Hard tissue height (BH) and soft tissue height (MH) were measured as an axial distance from the implant platform to the crest of hard tissue and the top of soft tissue on the labial/buccal aspect of implants (Fig. 1). Measurements of BW, MW, BH, and MH were done in CBCT scans from T1 to T3 for objected implants, respectively. For hard tissue resorption during the observation period, ΔBH was calculated as a numerical value difference of BH between T1, T2, and T3. The amount of mucosal tissue regression was also calculated in the same manner on ΔMH.

Two calibrated examiners measured the parameters 10 times of 10 randomly selected cross-sectional CBCT images which were acquired at T1 to assess the reliability and reproducibility of the measurement. The intra- and inter-examiner reliability of the measurements were expressed as intraclass correlation coefficient (ICC). Subsequently, a single examiner performed all the measurements and data collection. The examiner blindly examined and conducted measurement on cross-sectional CBCT images of objected implants.

### Classification of peri-implant hard and soft tissue

**Fig. 2:**
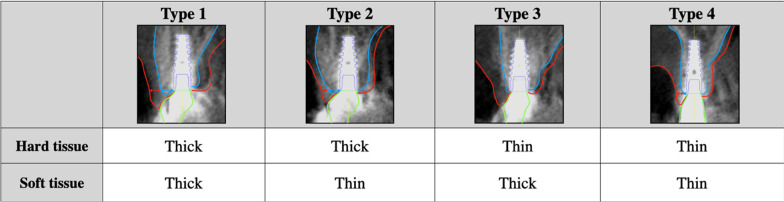
Representative CBCT images for classification of implants according to thickness of labial/buccal hard and soft tissue width. Cutoff thresholds were determined 1.6 mm for hard tissue, 2.5 mm for soft tissue. Implants with thick hard and soft tissue were classified as Type1, those with thick hard tissue and thin soft tissue were as Type 2, those with thin hard tissue and thick soft tissue were as Type 3, and those with thin hard and soft tissue were as Type 4

Subjected implants were classified into four groups according to the thickness of peri-implant hard and soft tissue on the labial/buccal aspect of implants. Implants with thick hard and soft tissue were classified as Type 1, those with thick hard tissue and thin soft tissue were classified as Type 2, those with thin hard tissue and thick soft tissue were classified as Type 3, and those with thin hard and soft tissue were classified as Type 4 (Fig. 2).

The cutoff thresholds for buccal/labial hard and soft tissue thickness were determined using receiver operating characteristic curve (ROC curve), 1.6 mm for buccal/labial hard tissue thickness, and 2.5 mm for buccal/labial soft tissue thickness. The ROC curve was drawn according to the buccal/labial thickness at the final superstructure installation as a continuous variable and the presence or absence of temporal resorption of the height of hard and soft tissues as a binary variable. The presence or absence of resorption of hard/soft tissue height over time was defined as 0.2 mm or more as the presence of resorption and less than 0.2 mm as the absence of resorption, based on previous reports [20–22].

### Statistical analysis

Statistical analysis was performed using SPSS Statistics ver.23 (IBM Japan, Tokyo, Japan). Intra-group comparisons of hard and soft tissue alteration were carried out using Friedman's test, followed by Wilcoxon signed-rank test with Bonferroni’s adjustment for multiple comparisons, and *P* < 0.017 was considered statistically significant. For inter-group comparisons of hard and soft tissue alteration, the Kruskal–Wallis test was carried out, followed by the Mann–Whitney *U* test with Bonferroni’s adjustment for multiple comparisons, and *P* < 0.008 was considered statistically significant.

## Results

### Clinical evaluation

Thirty-one patients met the inclusion and exclusion criteria. They underwent CBCT scan at the time of final prosthesis delivery (T1), 1-year follow-up (T2), and 2-year follow-up (T3). In all 31 patients, horizontal widths of labial/buccal hard and soft tissue on implant platform level were measured on cross-sectional CBCT images at the final prosthesis delivery (T1). No patient dropped out during an observational period of the present study, and 45 implants were included in the final analyses. The mean age of all patients was 57.5 years, range 19–74. No complications were reported during follow-up. All 45 implants were functioning adequately at 1-year and 2-year follow-ups.

### Classification of peri-implant hard and soft tissue

Forty-five implants were classified into four groups according to peri-implant hard and soft tissue width on the labial/buccal aspect of the implant. Eleven implants were classified as Type 1, 14 as Type 2, 10 implants were classified as Type 3, and 10 implants were classified as Type 4. The baseline demographic parameters are shown in Table 1. None of the parameters were statistically different among the four groups.

**Table 1:** Baseline demographic parameters for 45 implants

	Type 1	Type 2	Type 3	Type 4	*P* value
Number of implants	11	14	10	10	
Sex^a^					
Male	3	3	5	3	0.44
Female	7	10	4	5	
Age (year)^b^ Mean (min–max)	57 (42–74)	59 (41–74)	58 (19–74)	59 (19–74)	0.75
Region^a^					
Central incisor	4	6	3	4	0.31
Lateral incisor	6	3	4	2	
Canine	0	1	2	1	
First premolar	0	1	1	2	
Second premolar	1	3	0	1	
Clinical procedure^c^					
Implant placement only	4	4	0	1	0.07
Implant placement with GBR	3	4	5	9	
Implant placement with GBR/CTG	3	3	3	0	
Immediate placement	1	3	2	0	
BW (T1) (mm)^d^	3.2	2.7	1.3	1.3	< 0.01
MW (T1) (mm)^d^	2.8	1.8	3.2	2.2	< 0.01
Observational period (months)^b^					
T1–T2	11.9 ± 2.1	12.2 ± 1.8	14.1 ± 2.1	13.0 ± 1.6	0.35
T2–T3	13.3 ± 2.1	13.9 ± 2.4	13.0 ± 1.7	14.2 ± 2.6	0.56

None of the parameters were statistically significant among 4 groups other than BW (T1) and MW (T1)

^a^Fisher’s exact test; significance level of 0.05 was used

^b^Student’s *t* test; significance level of 0.05 was used

^c^*χ*^2^ test; significance level of 0.05 was used

^d^Kruskal–Wallis test; significance level of 0.05 was used

### Radiographic evaluation

The high intraclass correlation coefficient (ICC) was achieved (0.82–0.99; Table 2). Alteration of BH of 4 types during 2-year observation is shown in Table 3. BH decreased in all types from T1 to T3. BH decreased significantly in all types from T1 to T2. BH of Type 2 and Type 4 decreased significantly from T2 to T3 (*p* = 0.002 for Type2, *p* = 0.005 for Type 4). Alteration of MH of all four groups during 2-year observation is shown in Table 4. MH decreased in all four groups from T1 to T3. MH declined significantly in all types from T1 to T2. MH of Type 2 and Type 4 decreased significantly from T2 to T3 (*p* < 0.001).

**Table 2:** Intra- and inter-examiner differences between two examiner intra-class correlation coefficients (ICCs) for radiographic measurements

Measurement	Intra-examiner ICC (*n* = 10)	Inter-examiner ICC (*n* = 10)
BW (T1)	0.990	0.990
MW (T1)	0.969	0.821
BH (T1)	0.956	0.994
MH (T1)	0.973	0.972

**Table 3:** Alteration of BH of 4 groups during 2-year observation

	Type 1			Type 2			Type 3			Type 4		
	Median	Min	Max	Median	Min	Max	Median	Min	Max	Median	Min	Max
T1 (mm)	2.8	1.2	3.8	2.3	0.9	4.3	1.0	− 0.1	2.5	1.2	0	2.6
T2 (mm)	2.8	1.1	3.7	2.2	0.7	4.0	0.9	− 0.1	2.4	0.8	− 0.6	2.2
T3 (mm)	2.7	1.1	3.3	2.1	0.3	4.0	0.9	− 0.1	2.4	0.5	− 1.1	1.7

**Table 4:** Alteration of MH of 4 groups during 2-year observation

	Type 1			Type 2			Type 3			Type 4		
	Median	Min	Max	Median	Min	Max	Median	Min	Max	Median	Min	Max
T1 (mm)	4.7	2.7	6.3	4.3	2.7	6.0	4.5	1.8	5.0	3.0	0.8	4.3
T2 (mm)	4.7	2.6	6.1	4.1	2.5	5.7	4.4	1.8	4.8	2.4	0.3	3.8
T3 (mm)	4.7	2.6	6.1	4.0	2.4	5.6	4.4	1.8	4.6	2.1	0.1	3.4

**Fig. 3:**
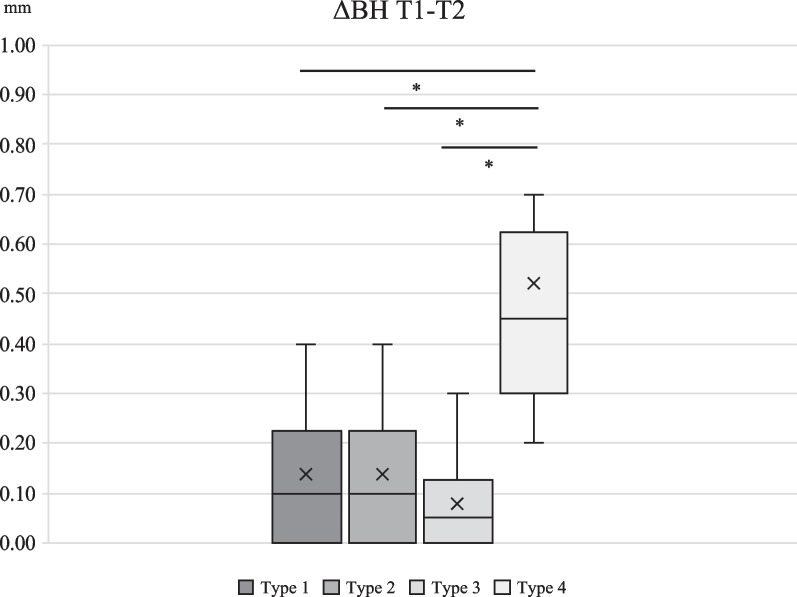
Inter-group comparison of numerical change of BH from T1 to T2. ΔBH for Type 4 was significantly greater than that of Type 1, 2 and 3 (*p* < 0.001)

**Fig. 4:**
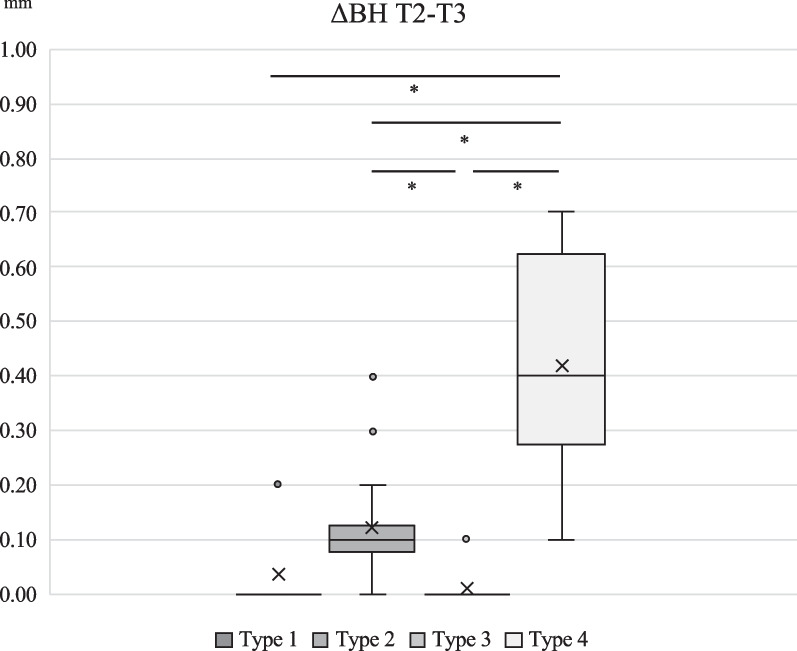
Inter-group comparison of numerical change of BH from T2 to T3. ΔBH for Type 4 was significantly greater than that of Type 1, 2 and 3 (*p* < 0.001). There was a statistically significant difference between Type 2 and Type 3 (*p* = 0.002)

**Fig. 5:**
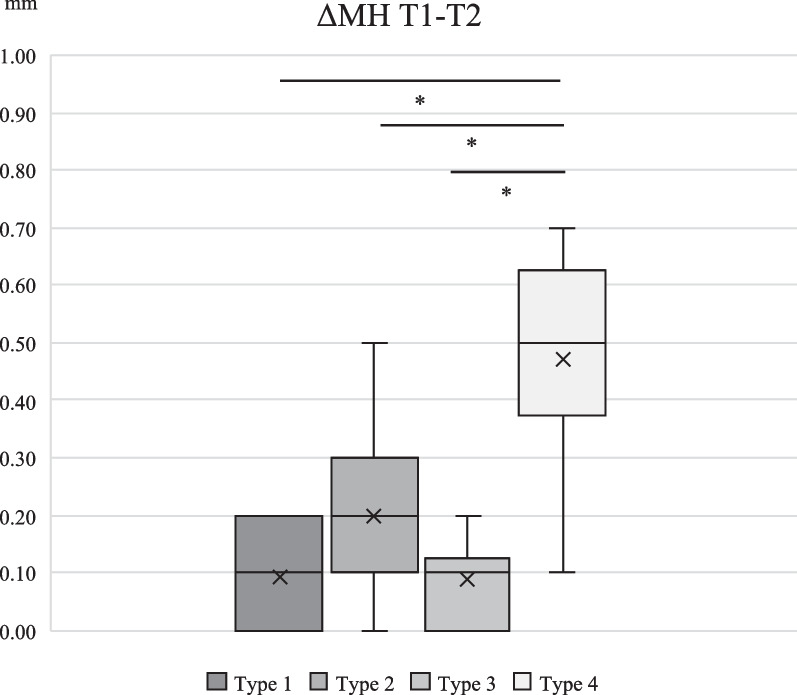
Inter-group comparison of numerical change of MH from T1 to T2. ΔMH for Type 4 was significantly greater than that of Type 1, 2 and 3 (*p* < 0.001)

**Fig. 6:**
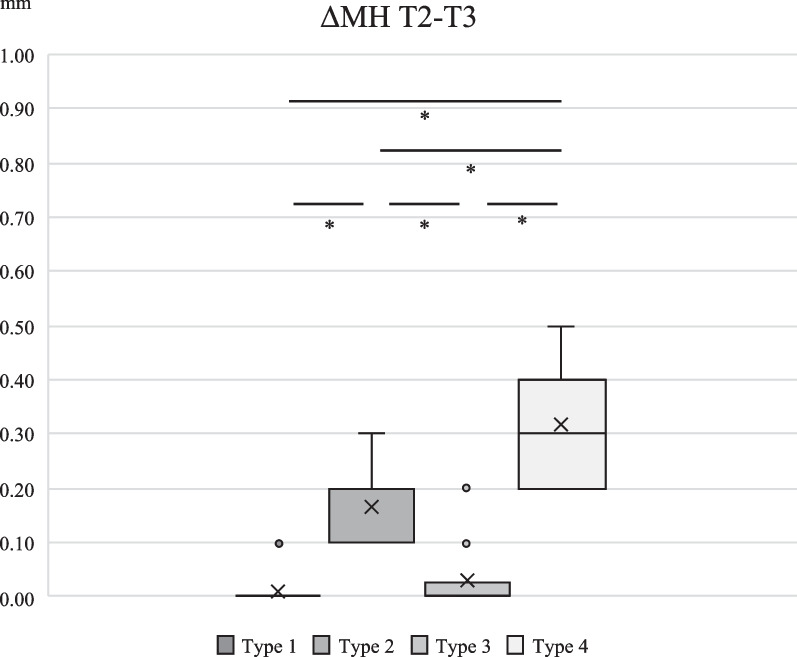
Inter-group comparison of numerical change of MH from T2 to T3. ΔMH for Type 4 was significantly greater than that of Type 1, 2 and 3 (*p* < 0.001). There were statistically significant differences between Type 1 and Type 2, Type 1 and Type 3 (*p* < 0.001)

Inter-group comparison of numerical change of BH and MH during 2-year observation is shown in Figs. 3, 4, 5 and 6. For the first half of the observational period, both ΔBH and ΔMH of Type 4 were significantly greater than the other three groups (*p* < 0.001). For the latter half of the observational period, both ΔBH and ΔMH of Type 4 were significantly greater than that of the other three types (*p* < 0.001). ΔBH of Type 2 was significantly greater than that of Type 3 (*p* = 0.002), and ΔMH of Type 2 was significantly greater than Type 1 and 3 (*p* < 0.001).

## Discussion

The present 2-year prospective study evaluated how peri-implant labial/buccal hard and soft tissue alter in 2 years by observing peri-implant labial/buccal hard and soft tissue thickness. To the best of the authors’ knowledge, no clinical studies have simultaneously investigated alterations of both peri-implant hard and soft tissue.

In the present study, the cutoff threshold was set at 1.6 mm for peri-implant labial/buccal hard tissue and 2.5 mm for peri-implant labial/buccal soft tissue. There are many reports that labio-palatal thickness of hard tissue should be 1.5 to 2.0 mm to reduce the risk of circumferential exposure of implant body neck. They reported that approximately 1.5 mm of dish-shaped bone resorption occurs around the neck of the implant body in 1 year after trans-mucosal structure placement [2–4]. These theories focused mainly about peri-implant hard tissue alteration around non-platform switching implants (NPS). On the other hand, it was also reported that platform switching (PS) implant is less likely to induce peri-implant tissue migration toward the apical direction since thicker soft tissue around implant body—abutment dimension should more likely be obtained in PS implant compared to NPS implant. In the present study, all implants observed were PS implants. In comparison between Type 1 with thick hard/soft tissue and Type 3 with thin hard tissue and thick soft tissue, there was no significant difference in hard and soft tissue resorption in 2 years. This result suggests that PS implants could tolerate an unfavorable environment for NPS implants when thick soft tissue is present around PS implants. Adequate labial/buccal soft tissue thickness has been reported in several studies and varies in amount; 1.0 mm [6], 1.5 mm [7], 2.0–3.0 mm [8]. As for esthetic consideration in mind, two studies suggests that 2.0 mm of labial/buccal soft tissue thickness is sufficient for preventing metallic color penetration of implant body and abutment [23, 24]. Linkevicius et al. conducted a comparative study; objected implants were subdivided into three groups according to vertical soft tissue thickness less than 2 mm, 2.5 mm, or thicker than 3 mm, then marginal bone loss in time was observed [16]. The authors pointed out that soft tissues were conventionally considered to be “thick” when they were 3 mm or more, which was determined out of an animal study, thus concluded that 2.5 mm or more in soft tissue thickness would be considered “thick”. The result of the present study showed that less peri-implant tissue resorption was observed in implants with thick soft tissue (2.5 mm or more) compared with those with thin soft tissue, which is partly consistent with the above-mentioned study. The threshold of soft tissues which determine them to be thin or to be thick has always been a controversy according to its variation of measurement methods and its difficulty in relating outcomes. Further studies should be conducted which provide conclusive scientific evidences for determining thick or thin tissues.

It was an interesting finding from the results of the current study that there was a slight difference between the first and the second period of observation from the aspect of tissue resorption. During the first period, from T1 to T2, the height of hard and soft tissues decreased significantly in any group, and implants with thin hard and soft tissues had significantly more resorption than the other three groups. This result supports the previous reports [5, 25] that thin tissue around implant is likely to cause tissue resorption over time. In the second period, from T2 to T3, implants with thick soft tissue such as Type 1 and 3 showed significantly less hard and soft tissue resorption than those with thin soft tissue, such as Type 2 and Type 4. Some studies reported that the presence of thick labial/buccal soft tissue prevents peri-implant tissue from resorbing in time [14–16, 26, 27], which is consistent with the result of the present study. It can be inferred from the result of the current study that peri-implant tissue might be stable after a certain amount of time after final prostheses placement, then as its stability may depend on the thickness of peri-implant soft tissue, implants with thin soft tissue would start to experience peri-implant tissue recession after the specific period of time. A clinical study which observed a ratio between peri-implant soft tissue height and width reported that thick soft tissue should be obtained around implants to maintain tissue stability due to its peculiar feature of peri-implant tissue [28]. Teeth and oral implants uniquely penetrate oral mucosa, connecting outer space to bone as inner structure which essentially should be completely separated from outer space. Reestablishment of supra-crestal tissue attachment followed by surgical and prosthetic interferences and its maturity can lead to tissue stability especially around natural dentition. Although it might be exaggerating to state that the mechanism of tissue stability is all the same for peri-implant tissue conditions, obtaining sufficiently thick tissue around implant supported prostheses should contribute to homeostasis of peri-implant tissue.

The major indices that determine the success of implant treatment are peri-implant bone resorption around the neck of implants and regression of the surrounding mucosa over time. Retraction of the surrounding mucous membrane with the progress of bone resorption in the neck of the implant body exposes the metallic color of the implant body surface to the oral cavity, causes esthetic complications, and creates a compromised environment that easily allows bacterial infection contamination on the implant surface. One of the critical criteria for successful implant treatment is that cervical bone resorption should be suppressed to less than 1.5 mm in the first year of function and less than 0.2 mm/year in the following period [29, 30]. When osseointegrated implant body communicates with the oral cavity, cervical bone resorption begins to occur along with the construction of biologic width around the implant [31]. It is reported that cervical bone resorption continues due to bacterial infection from the micro-gap at the occlusal load against implant–abutment junction and mechanical stress due to brushing pressure [32]. A study by van Eekeren et al. analyzed marginal bone loss around 78 implants in 33 patients for 1 year by sorting them into four groups by implant system and vertical mucosal thickness, showed a statistically significant difference in crestal bone change after 1 year of loading on peri-apical radiographs between bone level implants with thick mucosa and those with thin mucosa (*P* < 0.05) [33]. Another study by Linkevicius et al. analyzed 55 implants divided into three groups, those with thin mucosa (2.0 mm or less), those with medium mucosa (2.5 mm), and those with thick mucosa (2.5 mm or more), observing marginal bone loss in between implant placement and 1 year follow-up on peri-apical radiographs. Marginal bone loss after 1 year in function were 1.25 mm for implants with thin mucosa, 0.98 mm for medium mucosa, and 0.43 mm for thick mucosa, showing that statistically significant differences between thin and thick group (*P* < 0.001), medium and thick group (*P* = 0.0014) [16]. Both of these studies concluded that vertical mucosa thickness might be an important factor on crestal bone stability, as it was shown that when soft tissue thickness decreases, bone loss increases. These studies analyzed marginal bone loss on mesial and distal aspect of implants on peri-apical radiographs and labial/buccal bone alteration in time cannot be analyzed. Plus, mucosa thickness at baseline was measured in vertical dimension. Peri-implant tissue surrounds implant–abutment connection three-dimensionally, not only vertically but also bucco-lingually. Since it has not been scientifically evident whether vertical tissue thickness or horizontal tissue thickness is more important for marginal bone stability around implants, further studies should be carried out to clarify the importance of dimensional existent of surrounding tissue around implants.

The present study indicates that implants with thicker labia/buccal peri-implant soft tissue would show less peri-implant tissue resorption in time. The objected implants include those placed in sites utilizing procedures, such as simultaneous GBR, GBR and CTG or immediate implant placement. It is indicated by several articles that hard tissue around implants which underwent simultaneous GBR would likely resorb compared to native bone, while soft tissue acquired by CTG would likely to maintain its volume around both natural dentition and implants. With these facts in mind, it would be another perspective to study on how peri-implant tissue alters in time by categorizing implants according to surgical procedures. However, specifically on the current study, the alteration of peri-implant tissue by subdividing objected implants with tissue width, how strongly surgical procedures would affect on results remains unclarified. Therefore, one should be bear in mind that results of the current study might be influenced by various factors.

In accordance to every clinical study, the present study has several limitations. First of all, it is desirable that a clinical study should eliminate multi-factorial effect as much as possible. In the present study, although there were no significant differences in demographic parameters between observed four groups, factors such as the presence of keratinized mucosa [34], oral hygiene [35], and the numbers of abutment removal [36] which have been reported as involving factors for the stability of peri-implant tissues, were not included in the evaluation criteria. Therefore, ideally, a clinical study which conducts multivariate analyses including those factors taken into consideration should be carried out with a larger sample size. Second, the observation period is limited within 2 years after final prostheses placement. It is well-reported that peri-implant tissue recession could occur up to 5 years after implant plcement [37]. Thus, further clinical studies which follow a longer term after treatment is in need.

## Conclusions

The present study prospectively analyzed the influence of labial/buccal tissue morphology around implants on chronological bone loss and soft tissue recession after final prostheses placement for 2 years. Results indicate that thick soft tissue on the labial/buccal aspect of the implant could play an essential role in preventing both hard tissue resorption and soft tissue recession over time. However, it remains inconclusive how peri-implant soft tissue influences peri-implant tissue preservation. Clinical trials are needed to explore the effect of soft tissue phenotype on the long-term outcome of peri-implant tissue.

## Data Availability

The data sets generated and analyzed during the current study are available from the corresponding author on reasonable request.
